# Learn to Bet: Using Reinforcement Learning to Improve Vehicle Bids in Auction-Based Smart Intersections

**DOI:** 10.3390/s24041288

**Published:** 2024-02-17

**Authors:** Giacomo Cabri, Matteo Lugli, Manuela Montangero, Filippo Muzzini

**Affiliations:** Department of Physics, Informatics and Mathematics, University of Modena e Reggio Emilia, 41125 Modena, Italy; giacomo.cabri@unimore.it (G.C.); 283122@studenti.unimore.it (M.L.); filippo.muzzini@unimore.it (F.M.)

**Keywords:** deep reinforcement learning, smart city, intersection management, auctions, connected vehicles, autonomous vehicles

## Abstract

With the advent of IoT, cities will soon be populated by autonomous vehicles and managed by intelligent systems capable of actively interacting with city infrastructures and vehicles. In this work, we propose a model based on reinforcement learning that teaches to autonomous connected vehicles how to save resources while navigating in such an environment. In particular, we focus on budget savings in the context of auction-based intersection management systems. We trained several models with Deep Q-learning by varying traffic conditions to find the most performance-effective variant in terms of the trade-off between saved currency and trip times. Afterward, we compared the performance of our model with previously proposed and random strategies, even under adverse traffic conditions. Our model appears to be robust and manages to save a considerable amount of currency without significantly increasing the waiting time in traffic. For example, the learner bidder saves at least 20% of its budget with heavy traffic conditions and up to 74% in lighter traffic with respect to a standard bidder, and around three times the saving of a random bidder. The results and discussion suggest practical adoption of the proposal in a foreseen future real-life scenario.

## 1. Introduction

The Internet of Things (IoT) [1] is rapidly expanding, offering new possibilities in almost any aspect of our daily life by “making things smart”. In this work, we concentrate on urban mobility, as it plays a critical role in shaping urban life. However, this crucial element is often beset with challenges that significantly impact the overall urban experience. The intricate web of transportation systems, including road networks and public transit, not only influences the efficiency of daily commutes but also has far-reaching implications on environmental sustainability, air quality, and the overall quality of life for citizens. The delicate balance required to ensure smooth urban mobility is often disrupted by factors such as traffic congestion and the environmental toll of excessive vehicular activity. As cities continue to grow and evolve, addressing the complexities of urban mobility becomes imperative for fostering sustainable, accessible, and livable urban environments. In this context, smart mobility has the potential to enhance street safety as well as the driving experience in smart cities. We can imagine a future in which our streets will be populated by autonomous vehicles that not only pick the quickest route from point A to B but also factor in safety, route cost, driver preferences, current traffic, even the proximity of cultural spots, and so on [2].

To reach this goal, the implementation of smart mobility requires not only a robustly connected infrastructure connecting vehicles and their environment but also appropriated algorithms and methods for effective management and coordination of vehicle movements. For example, semantic traffic understanding might be exploited to define the number of vehicles in a lane by means of vision-based algorithms [3]. Indeed, road traffic is an extremely complex system and intersections, in particular, pose a significant challenge: a delicate balance is required between assigning priority in a fair way and the flexibility needed for emergency situations. Traditional solutions involve traffic yield rules, traffic lights, and roundabouts that are used to address the unpredictable nature and reaction times of human drivers, as well as the characteristics of the intersections (e.g., yielding to larger and more heavily trafficked lanes). These solutions, however, are not always the best-performing ones, as demonstrated by the frequent traffic jams occurring in our cities.

With the advent of autonomous and connected vehicles, and the possibility to equip them with intelligent systems, new solutions for intersection management can be devised to make the most of the potential of new technologies. In particular, it is possible to precisely instruct vehicles on their movements, predict their behavior and position, and make them communicate with each other and with city infrastructure to make informed decisions.

In this paper, we place ourselves in a scenario in which vehicles are autonomous and connected (falling under Level 5 automation [4]) and we assume that intersections are managed by means of auctions [5]: vehicles approaching an intersection place a bid to win the right to go through the intersection, and only the winner of the auction (i.e., the one placing the highest bid) is granted this right. The problem is to determine how to define the amount of the bids so that trip times are fair for all vehicles, to avoid vehicles getting stuck at an intersection because they run out of budget, and to make sure that traffic flows even in heavy traffic conditions.

We propose a vehicle bidding strategy based on reinforcement learning that is to be used when vehicles travel on frequently traveled routes: vehicles learn how to bet in order to grant themselves the right to cross intersections and to save a percentage of their budget for successive trips. We tested the proposal of the paper with an extensive set of experiments that show that the proposal is effective, robust, and usable in real life scenarios. Indeed, the percentage of the saved budget might be very large, at the expense of a small delay in arrival time at the destination. Moreover, the trained solution works well even under conditions that differ from the ones used for the training.

The paper is organized as follows: the rest of this section presents related work on autonomous vehicles coordination, intersection management systems using auctions and reinforcement learning; Section 2 presents the scenario we place ourselves in, the proposal of this paper for the bidder exploiting reinforcement learning and the experimental assessment; Section 3.2 presents and discuss the results of the experiments and future works; finally, conclusions are drawn in Section 4.

### 1.1. Related Work

The coordination of autonomous vehicles can be tackled from different points of view and by different solutions [6]. In connection with the work we carried out and presented in this paper, we report on general approaches in the field (Section 1.1.1), then we focus on the management of intersections by means of auctions (Section 1.1.2) and finally we present some related work about learning (Section 1.1.3).

#### 1.1.1. General Coordination of Autonomous Vehicles

There are different research efforts that address the design of the smart cities [6] in which autonomous vehicles will have to interact with each other and with their surrounding environment, requiring the vehicles (or, better, their software) to make decisions and take actions.

Even if the exploration of such topics is not new, it is only in recent times that newly introduced computer accelerators have achieved the requisite Size, Weight, and Power (SWaP) characteristics, enabling experimentation on medium to large-scale fleets of intelligent vehicles within specifically equipped urban areas [7,8].

Pinciroli et al. [9], drawing from their expertise in autonomous robots, draw attention to the distinction between smart devices and autonomous vehicles in the scenario of navigation. They emphasize that smart devices have limited capabilities of interaction with the physical world, while robots in general, and autonomous vehicles in particular, have the capability to engage with, and act upon, the surrounding environment through the utilization of sensors and actuators. Their proposed solution centers on a *swarm language construct* allowing the categorization of robots into swarms and the assignment of tasks to these swarms. Their primary objective is to ensure *re-usability* and *predictability* of the coded behavior, addressing crucial issues arising in the autonomous driving domain.

Murthy et al. [10] present a simulated environment designed for the aim of having cars travel on a highway and self-organize into platoons, so that they might reduce fuel consumption through drafting off one another. This approach aligns with the preceding one, emphasizing the pursuit of reusability and predictability within a specific application scenario.

Other research focuses on the capabilities of the smart city to manage different common urban problems such as parking [11,12]. For example, in [13] the smart city infrastructure is exploited to monitor the availability of parking spots, and the authors propose a mobile app that provides the parking spot information to the final user. In [14], the possibility of reserving a parking spot is introduced and in [15] this feature is extended to permit the booking some days in advance. In [16] the system ensures that a booked parking spot will be occupied only by the user that has booked that spot. On the other hand, in [17] the system does not ensure that a vehicle will find a free spot when it reaches the selected parking area but, in the case that there is not an available spot, the system can suggest the closest parking area with a free spot. In [18], a centralized system is proposed to manage the parking area taking into consideration the ideas above. Moreover, they consider an environment in which connected and non-connected vehicles coexist. Finally, in [19], the authors propose a city-wide parking guidance system that is able to suggest active recommendations and real-time adjustment while the vehicle is roaming. They use a generative network to generate parking lots without data. This network is trained using real data capturing the size of the parking area concerning the surrounding environment.

Lastly, the smart city infrastructure is exploited to suggest alternative routes to vehicles to reduce traffic congestion. For example, in [20], the authors propose an algorithm to suggest the best route based on the actual traffic situation. A vehicle continuously asks for the actual best route to react to traffic changes. Other works, instead, focus on extraordinary events like accidents [21] or bridge collapses [22]. In the first, the authors propose a system that suggests an alternative route to avoid accidents sites and reduce the emergency vehicle response by asking vehicles to flush the emergency vehicle’s route. In the second, the authors propose a system in which users can be informed about a disruptive event and can reschedule their trips and activities. Moreover, the authors analyze the impact on traffic of these changes.

#### 1.1.2. Intersection Management by Auctions

The proposal presented in this paper uses auctions to manage intersections. One of the first contributions in the domain of auction-based intersection management that has significantly shaped subsequent research endeavors is presented in the work authored by Carlino, Boyles, and Stone [5]. This work systematically addresses vehicles value of the time, employing a wallet system designed for automated bidding. This bidding mechanism is based on diverse parameters, including trip characteristics, driver-specified budget considerations, and the remaining distance to the destination. Furthermore, the authors extend their study to the optimization of the traffic ecosystem.

A more complex proposal is provided by Schepperle and Böhm [23], presenting a two-step second-price sealed-bid auction mechanism [24] to address intersection management: the vehicle with the highest bid wins but it has to pay only for the second-highest bid amount. In the initial phase, only vehicles with the capability to traverse intersections participate, while the subsequent phase expands bidding privileges to vehicles occupying the second position in the lane. In our work, we also consider other vehicles in the lanes than the first one, but not just the second in line: all the vehicles in line might contribute to the bid of the one bidding to get through the intersection, and the auction resolves in one phase only.

Vasirani and Ossowski [25] present an alternative solution for urban intersection management, employing a reservation-based intersection control model together with rules inspired by market dynamics. The authors scrutinize two distinct scenarios, one involving only one intersection and the other one with a network of intersections, with the aim of understanding the effectiveness of the policy-driven slot reservation mechanism and the impact of a traffic assignment strategy influenced by competitive markets on drivers’ route selections.

In our work, we placed ourselves in the context of auction intersection management by keeping the single-step approach without reservation, and we made an effort to improve the bidding strategies by adding learning capabilities to vehicles with the aim of making them save budget on routes traveled frequently.

#### 1.1.3. Learning

Over the past few years, *reinforcement learning* techniques have been integrated into theautonomous driving context [26]. An example includes improving the interaction between the driver and the vehicle [27]. Moreover, reinforcement learning is used in the management of road infrastructures such as traffic signals [28]. As an example, in [29], a dynamic approach to intersection management is employed. A manager is trained using *Q-Learning* [30], enabling adaptive selection of the most effective method to facilitate traffic flow near the intersection at any given moment. In [31] Joo et al. use Q-learning to train a traffic light manager whose goal is to maximize the throughput of the crossroad while also minimizing the standard deviation of the queue’s length. A popular variant of Q-learning is called *Deep Q-Learning*, proposed by Google DeepMind in one of their milestone papers [32]. It solves the problem of *curse of dimensionality* by using neural networks instead of a matrix to store the learning information.

P. Karthikeyan et al. [33] propose a complex manager trained with Deep Q-learning made of three different models (*Break safe control model*, *Intersection control model*, and *Priority assignment model*) whose continuous interaction handles the whole crossroad. The first ensures traffic safety by preventing collisions between vehicles, the second chooses the best crossing policy at each time to maximize efficiency, and the latter handles priority requests sent by the waiting vehicles to the manager. In [34] the concept of latency introduced by the network is integrated in the intersection manager. Using reinforcement learning, the system can learn a control policy that is able to deal with changes in communications latencies. It internally models the behavior of autonomous vehicles based on their latency, and adapts the control of these vehicles to avoid collisions.

A different approach is proposed in [35], by applying Multi-Agent Reinforcement Learning to control traffic signals, the system learns traffic lights’ green and red signal duration and optimizes traffic flow.

In another line of research, the focus is on the vehicle and not on the intersection manager. For instance, in [36], reinforcement learning is used to learn the maneuvers of a connected autonomous vehicle in unsignalized intersections. The goal is to learn to break, steer, and throttle to safely cross the intersection. In [37], vehicles learn the desired policy to cross the intersection without any prior information about the scenario and infer both the intentions of the adversarial vehicles and the type of intersection.

A summary of works in the field of intersection management is reported in Table 1.

Our work, unlike the cited works, focuses on auction-based intersection management, with the goal of learning the optimal bidding strategy that allows for lower bids without sacrificing much travel time.

## 2. Materials and Methods

We place ourselves in a scenario where all vehicles are autonomous and connected. In this context, they possess the capability to communicate with each other and with city infrastructure, eliminating the need for human intervention in driving. We propose a solution for budget management at intersections that are based on auctions (i.e., vehicles approaching an intersection participate in an auction to win the right to cross it) in which vehicles that frequently travel on the same route have the possibility to save some of their budget by learning how to properly place bids on the routine route.

In this section we first present the scenario and then the new proposal in detail.

### 2.1. Scenario

In this section, we present the scenario we work in and the assumptions we make.

*Street maps.* We assume that each street has only one lane per direction. When streets intersect we have an *intersection* (or crossing). Street maps can be represented as graphs: there is a node for each intersection and a directed edge (called *link* or *lane*) for each street connecting two crossroads.

*Auctions.* Vehicles participate in auctions when they are the first of their lanes at the intersection site, with the aim of winning the right to go through. To define the auction strategy we describe how bids are computed and placed, and how the controller states which lane wins the auction.

*Initial budget.* Each vehicle is assigned a trip *budget* and this is used to place bids. It is outside the scope of this paper to define how budgets are assigned to vehicles, as we think that this issue is more an administrative/political than technical one: local administrations might decide to assign budgets according to drivers’ conduct, ecological incentives, drivers’ average Km/yds traveled per year, and so on, or even decide to sell budget.

*Vehicle bidder* (or *bidder* for short). Vehicles place bids when they reach an intersection, i.e., when they do not have any other vehicle ahead in the lane. The bidder defines the vehicle bidding policy. We call *standard bidder* a bidder that adopts the policy defined in [5]: the bid is computed as $\frac{B}{crossroads}$, where *B* is the vehicle’s total trip budget and crossroads is the number of intersections the vehicle will encounter in its route, starting from the current position to the destination. The bidder also defines the vehicle *sponsorship* [38], i.e., when waiting in line, the vehicle might endorse the bid of the vehicle currently at the intersection site by tipping a small percentage of its available budget. This tip is computed as B·β, where β is a positive parameter that depends on the vehicle, always strictly smaller than 1.

*Auctioneer* (or intersection manager). Each intersection is equipped with a traffic manager that collects the bids coming from the vehicles that are at the front of their lanes and, eventually, the sponsorships. Then it proceeds to determine the winner of the auction.

*Auction winner.* The winner of the auction is the one with the total higher bid, computed as the sum of the vehicle bid and its sponsorships. Only the winner of the auction is given the right to cross the intersection and has to pay its bid, while the others will have to participate in the next auction again. To avoid starvation, we exploit budget *redistribution* [38]: the amount of the bet of the winner is redistributed among the participants of the auction that did not win. In this way, even a vehicle with a low budget and no (or small) sponsorship will eventually collect enough budget to win the auction.

### 2.2. The Proposal

We present vehicle bidding policy that allows a vehicle to save budget on a route that it frequently takes at a specific time of day, without significantly impacting its waiting time in traffic.

Through reinforcement learning the bidder can autonomously learn how to place smart bids and sponsorships in order to achieve its goal. More specifically, the bidder applies a “discount” on the bid or sponsorship that he would normally place (according to the description in Section 2.1). Experiments will show that applying discounts in a smart way leads the bidder to save a considerable amount of currency sacrificing negligible time.

### 2.3. Reinforcement Model

The proposed reinforcement learning model uses Deep Q-learning to teach the bidder how to manage the vehicle’s budget. The bidder is the *agent* attempting to maximize the *reward* over time, which is a feedback signal sent by the environment to the agent. It is positive if aligned with the agent’s desired behavior and negative otherwise.

Each vehicle waiting in a lane is given a *position* at each iteration, that is the number of vehicles it has ahead in the lane at the beginning/end of the iteration, and thus the vehicle at the front of the lane has position zero.

The reward *R* is a linear combination of two rewards, one relative to the vehicle’s position and the other to the bid discount:

- The *Position Reward* $R_{p}$ depends on the result of the vehicle’s bid or sponsorship. If the vehicle participates in an auction and wins, it gets a positive reward, if it loses it receives a negative one. Otherwise, if the vehicle sponsors another one it receives a reward proportional to its advancement in the lane;
- The *Discount Reward* $R_{d}$ is the fraction of the bid saved with the discount, with respect to the standard bid. High values are obtained if the bidder applies convenient discounts.

Equation (1) shows how *R* is computed:

(1) $$\begin{array}{cc} R_{p} & =\left\{\begin{array}{cc} w & \mathrm{if}\;\mathrm{the}\;\mathrm{vehicle}\;\mathrm{wins}\;\mathrm{an}\;\mathrm{auction},\\ \ell & \mathrm{if}\;\mathrm{the}\;\mathrm{vehicle}\;\mathrm{loses}\;\mathrm{an}\;\mathrm{auction},\\ P_{i}-P_{f} & \mathrm{if}\;\mathrm{the}\;\mathrm{vehicle}\;\mathrm{is}\;\mathrm{sponsoring}, \end{array}\right.\\ R_{d} & =1-d,\\ R & =\alpha R_{p}+\left(1-\alpha \right)R_{d}, \end{array}$$

where:

- $P_{i}$ is the initial position of the agent, i.e., the position at the end of the previous iteration;
- $P_{f}$ is the final position of the agent, i.e., the position at the current iteration;
- w>1 is a positive parameter representing the reinforcement signal when the vehicle wins an auction.
- ℓ<0 is a negative parameter representing the reinforcement signal when the vehicle does not win an auction.
- *d* is the multiplier returned by the bidder, related to a specific action *action* $a_{t}$. For example, a discount of 90% on a bid corresponds to a multiplier of 0.1 returned by the bidder. This translates to a *discount reward* ($R_{d}$) of 0.9, which is high;
- α is a tuning parameter used to balance the position reward and the discount reward.

### 2.4. Neural Network Architecture

We used a simple multilayer perceptron neural network. The input of the first layer is a state of the system $s_{t}$, represented as a tensor of *C* values as described in Equation (2):

(2) $$\begin{array}{cc} s_{t} & =\left[\begin{array}{c} x_{1t}\\ x_{2t}\\ \vdots \\ x_{Ct} \end{array}\right], \end{array}$$

where, *C* is the number of crossroads managed with auctions on the map, each entry $x_{it}$ is defined in Equation (3):

(3) $$x_{it}=\left\{\begin{array}{cc} \frac{L-P_{f}}{L}, & \mathrm{if}\;\mathrm{the}\;\mathrm{test}\;\mathrm{vehicle}\;\mathrm{is}\;\mathrm{waiting}\;\mathrm{at}\;\mathrm{crossroad}\;i\\ 0 & \mathrm{otherwise} \end{array}\right.$$

and *L* is the number of vehicles ahead in the lane.

This encoding allows the bidder to accurately determine its current intersection and roughly estimate its position in the queue of vehicles. The value *L* represents the length of the queue at the intersection ahead of the bidder and is used as a normalization factor to scale the values of $x_{it}$ between [0, 1], ensuring that the input stays in a controlled range. On the other hand, this scaling reduces the bidder’s understanding capabilities, as it doesn’t precisely know how many vehicles are present in its lane (*L* itself is not part of the input forwarded through the neural network).

The input layer is followed by 3 hidden dense layers, each one composed of 128 neurons. For each hidden layer we use the activation function *ReLU*. The output layer has eleven neurons, one for each possible discount that can be chosen by the bidder.

At each forward step *t*, the output neurons $\vec{p}$ will contain the Q-values of available actions. The model exploits the best action by choosing the index of the neuron containing the highest Q-value, or explores the action space by picking a random index. To compute the corresponding discount $d_{t}$, the chosen index is divided by 10, as in Equation (4):

(4) $$\begin{array}{cc} \; & a_{t}=\left\{\begin{array}{cc} \mathrm{argmax}(\vec{p}), & \mathrm{if}\;\mathrm{agent}\;\mathrm{is}\;\mathrm{exploiting},\\ \mathrm{rand}(0,10) & \mathrm{if}\;\mathrm{agent}\;\mathrm{is}\;\mathrm{exploring} \end{array}\right.\\ \; & d_{t}=\frac{a_{t}}{10} \end{array}$$

By performing action $a_{t}$, the bidder multiplies a bid or a sponsorship by the discount factor $d_{t}$. Finally, we use Adam [39] as the optimizer.

### 2.5. Training

The vehicle bidder has been trained using the Deep Q-learning algorithm described in Algorithm 1. The algorithm uses two neural networks *Q* and *T* to improve the overall training stability [40] and it is based on experience replay as shown in Algorithm 2. the weights of the *Q-network Q* are updated by using *MSE* (mean squared error) as the loss function, and are periodically copied in the *Target-network T*.
**Algorithm 1** Q-learning algorithm**Input** Untrained Q (Q-network) and T (Target network) **Variables** $a_{t}$: action executed at time *t*; $s_{t}$: state of the environment at time *t*; *R*: reward obtained by executing $a_{t}$ in state $s_{t}$; *M*: experience replay memory; *F*: training frequency; *K*: target-network update frequency; **Output**
Q,T: trained Q-network and Target network;
  1: samples←0▹ sample counter  2: t←0▹ training counter  3: **for** each bid or sponsorship made by test vehicle **do**  4:    Observe $s_{t}$;  5:    Pick a random $a_{t}$ with probability ϵ, else choose $a_{t}=\mathrm{argmax}\left(Q(s_{t})\right)$;  6:    Perform action $a_{t}$ (apply multiplier $d_{t}$ on either bid or sponsorship);  7:    Observe $s_{t+1}$ and reward *R*;  8:    *M*.append$\left(<s_{t},a_{t},s_{t+1},R>\right)$;  9:    samples←samples+1;10:    **if** samples==F **then**11:        samples←0;12:        t←t+1;13:        Experience replay (Q, M);▹ train *Q* using Algorithm 214:        **if** t==K **then**15:           t←0;16:           Update target network;▹ copy weights of Q in T17:        **end if**18:    **end if**19:    $s_{t}=s_{t+1}$;20: **end for**

**Algorithm 2** Experience replay**Input** *Q*: Q-network; *M*: experience replay memory, containing tuples $<s_{t},a_{t},R,s_{t+1}>$; **Variables** *b*: batch size; γ: discount factor; η: learning rate; T: Target network;

 1:**if** len(M) > *b* **then** 2:    *L* = randomly sample *b* tuples from M; 3:    **for** $<s_{t},a_{t},R,s_{t+1}>$ ∈L **do** 4:        $\vec{p},\vec{t}=Q\left(s_{t}\right)$ 5:        $\vec{h}=T\left(s_{t+1}\right)$ 6:        $\vec{t}\left[a_{t}\right]=R+\gamma \max\vec{h}$ 7:        *Q*.fit $\left(\mathrm{prediction}=\vec{p},\;\mathrm{target}=\vec{t},\;\mathrm{epochs}=1,\;\mathrm{learning}\;\mathrm{rate}=\eta \right)$ 8:    **end for** 9:
**end if**



The experience replay memory *M* contains tuples of the form $<s_{t},a_{t},R,s_{t+1}>$, where the $s_{i}$s are the states of the environment at time i∈{t,t+1}, $a_{t}$ is the action taken at time *t*, and *R* is the reward obtained by executing action $a_{t}$ in state $s_{t}$ computed using Equation (1).

Hyperparameter *F* represents the training frequency, meaning that the neural network *Q* is trained only once each *F* samples is stored in *M*. This introduces more variability at each training step and makes the simulation faster. Hyperparameter *K* represents the target network update frequency, i.e., after how many training time steps the target network *T* is updated.

We use a variant of the ϵ-greedy strategy [41] in which ϵ varies to favor exploration in the early stages of the simulation and exploitation in the later stages. Given the number of samples len(M) in experience replay memory *M*, ϵ is defined as in Equation (5):

(5) $$\begin{array}{cc} \epsilon & =\left\{\begin{array}{cc} 0.3 & \mathrm{if}\;0<len\left(M\right)<\frac{E}{2}\\ 0.2 & \mathrm{if}\;\frac{E}{2}<len\left(M\right)<E\\ 0.1 & \mathrm{otherwise} \end{array}\right. \end{array}$$

where *E* is a threshold value.

### 2.6. Experiments

We tested the proposal in a simulated environment using the urban mobility simulator SUMO [42].

We chose SUMO among the plethora of urban traffic simulators present in literature [43], due to its open-source nature. Another commonly used open source simulator is MATSim [44]. Many works conclude that there is not a simulator that clearly outperforms the other [45,46] but we choose SUMO for its Python interface that allows us to easily integrate the RL tools.

The hardware on which we performed both the training and testing has the following specifications: Intel(R) Core(TM) i5-8400 2.80 GHz CPU (Intel, Santa Clara, CA, USA) NVIDIA GeForce GTX 1060 6 GB GPU (Intel, Santa Clara, CA, USA) and 8 Gb of RAM.

We performed a set of preliminary experiments in order to train the model under different configurations and select the best- performing one. The latter will be then be used in the experimental phase to show the effectiveness and robustness of the proposed bidder.

In all experiments, there will be one test vehicle always traveling the same route and adopting a specific bidder, while all other vehicles adopt the standard bidding strategy introduced in Section 2.1.

#### 2.6.1. Set Up

Both *Preliminary Experiments* and *Experiments* were performed using a Manhattan-style map of 5 × 5 streets and 25 intersections. This size of a map is adequate for around 120 vehicles, meaning that roads are neither not too congested nor too empty. The innermost 9 intersections have four lanes competing for crossing and are managed using the auction systems proposed in Section 2.1.

Each run of an experiment has a fixed number of vehicles (*VS*), all of which are able to participate in auctions. Vehicles follow circular routes and, when one ends, they start again with a new one. At the beginning of each route, their budget is reset to a default initial budget IB. This ensures a fair and consistent financial starting point for every iteration, allowing a balanced experience across all vehicles in the simulation.

For the test vehicle, β and *v* are fixed among both *Preliminary Experiments* and *Experiments*, and they have been set to 0.10 (average sponsorship) and 4 (slow), respectively. These choices are meant to put the test vehicle in a slightly harder situation than an average vehicle.

Other parameters related to simulations and auctions (including β and *v* for non-test vehicles, *IB* for the test vehicle, and *VS*) may vary depending on the experiment. In Table 2, a summary of the parameters and their respective ranges of values is provided.

Speed values are expressed in the simulator’s unit of measurement. The absolute value of speed is not crucial. Rather, relative speed is, and it is significant that some vehicles are nearly as twice as faster than others. This adds variability to the simulations, emulating what typically occurs in city traffic. The number of vehicles depends on the size of the map and has been selected in order to avoid two extreme situations: vehicles roam on empty streets (any solution will work well), and vehicles are in deadlock (no solution will work). As for the sponsorship parameter, referring to [47], in which vehicles could allocate up to 25% of their budget for sponsorships, here vehicles adopt a slightly more conservative strategy: we allow vehicles to spend maximum 15% of their available budget. This choice is driven by the fact that in our experiments, we have a larger number of vehicles (up to 150) compared to the previously mentioned work (at most 120).

Table 3 summarizes and reports the values of all parameters, hyperparameters, and thresholds used through simulations for the reinforcement learning algorithm. Values have been tuned by extensive simulations to determine best-performing ones.

#### 2.6.2. Preliminary Experiments

We train the model under different configurations, resulting in different trained models. The best performing one will then be used for the experiments; for this purpose, we are interested in the model that shows the best trade-off between saved budget and waiting times with more emphasis on saved budget. With this aim, we select one test vehicle that uses the reinforcement learning bidder and compare its performances with that of an identical vehicle that adopts either the standard bidder or a random behavior for what concerns its discount policy.

Each configuration has two types of vehicles:

- *Routine Vehicles* perform the same routine every day (same trip at the same time), e.g., they simulate drivers going to work in the morning and back home in the afternoon. The parameters associated with these vehicles do not change among simulations.
- *Random Vehicles* do not have a repetitive or predictable behavior, e.g., they simulate vehicles that are passing through the block or city. Parameters β and *v* associated with these vehicles are initialized randomly at each simulation.

Each configuration is associated with a percentage of random vehicles roaming the map, where the percentage is chosen in the set {0%,10%,20%,30%}, and one single test vehicle. All vehicles, with the exception of the test vehicle, adopt the standard bidding strategy described in Section 2.1. Each configuration is trained and evaluated while varying the number of vehicles on the map, ranging from 80 to 150.

We measure the performance of a trained configuration by means of the following metrics:

- Saved Budget percentage (*S*), i.e., how much budget is left at the end of a trip, given as the percentage of the initial budget.
- Waiting times. In particular:
  – Traffic Waiting Time (*TWT*): the time spent waiting in lanes, i.e., from when a vehicle enters a lane until it exits the lane. The counter related to each vehicle measuring its *TWT* is updated only when its speed is approaching zero (meaning it is waiting in traffic).
  – Crossing Waiting Time (*CWT*): the time spent waiting at the intersection site as the first vehicle of the lane, i.e., from when the vehicle becomes the first vehicle to when it crosses the intersection.

For each configuration, we compute mean and standard deviation for *S*, *TWT*, and *CWT* over 10 runs, where vehicles follow the same routes but might vary their maximum speed *v* and β.

The average of saved budget over *N* trips is computed as in Equation (6):

(6) $$S_{a}=\left(\frac{1}{N}\sum_{i}^{N}\frac{IB-FB_{i}}{IB}\right)\times 100,$$

where $FB_{i}$ is the final budget left at the end of trip number *i*, and the number *N* of trips depends on the length of the simulation (it is 8 on average in our experiments).

We compare the performances of such a test vehicle with that of a vehicle that has exactly the same parameters and the same behavior (e.g., same routes) as the tested vehicle but that uses (1) the standard bidder, and (2) a *random bidder* that randomly chooses a discount to apply to each bid or sponsorship made by the test vehicle.

Results presented in Section 3.1 show that the best-performing configuration is the one trained with 20% of random vehicles and this is the selected one used for the experiments.

#### 2.6.3. Experiments

We performed a set of exhaustive experiments using the best model retrieved from preliminary experiments. Results are described and discussed in Section 3.2. Note that the model is not trained again, and the aim is to test the robustness of that model in scenarios that are different from the training one. This is a crucial aspect of the proposal, since in the real world conditions might vary with respect to those in which the model has been trained and it is important that a model performs well also in those situations. If it does not, it is of limited use.

We performed three experiments by varying: (1) the number of vehicles (*VS*) on the map, to show the robustness of the proposal when the traffic is more variable and heavier than the scenario in which the model was trained; (2) the ratio of random vehicles, to show the robustness against unpredicted vehicle behavior, and (3) the initial budget *IB* of the test vehicle, to show robustness against budget assignation choices. We measured waiting times and savings again, as described in Section 2.6.2.

## 3. Results

In this section, we report the results of both preliminary experiments and experiments.

### 3.1. Preliminary Experiments

Four models were trained in different configurations, by varying the percentage of random vehicles on the map. We then tested each trained model by varying the total number of vehicles on the map. Vehicles start each trip with a fixed budget that is the same for all.

We compared the performances, by means of saved budget and waiting times, of the test vehicle adopting the learning strategy against an identical vehicle adopting either the standard bidding strategy or a random discount policy for bidding and sponsorship. As for saved budget, we do not report results for the standard bidder strategy, because a vehicle adopting this approach will always have drained all its budget by the end of the route.

The trained bidder is always able to save more budget than the random bidder (and of course, than the standard bidder) even in different traffic conditions (i.e., number of vehicles) with respect to the training conditions. Conversely, we will see that the largest savings occur when the trained vehicle is slightly slower than its identical copies adopting the other strategies. This shows that there is a trade-off between the saving capacity and the duration of the trip. As the differences in budget savings are greater than the differences in trip times, we focus on the former and we will select the model leading to larger savings.

As a result of the preliminary experiments, we selected the model in which the bidder was trained with 20% of random vehicles.

In the following portions of this section, we show detailed results for the tested configuration.

Scenario 1: percentage of random vehicles is 0%.

**Figure 1:**
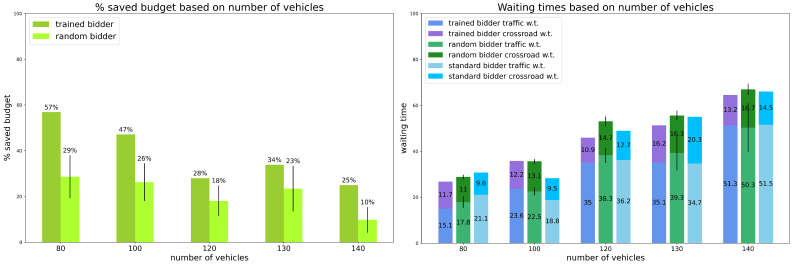
Preliminary Experiments. Results for scenario 1: percentage of random vehicles is equal to 0%. Vertical black lines represent standard deviations. Standard bidder’s savings are not reported because saved budget is always 0%.

The first model was trained with no random vehicles, and the results are reported in Figure 1.

We observe that the trained bidder shows a saving of up to 57% of the initial budget when there are 80 vehicles on the map, a situation that simulates low-traffic conditions. Saving decreases when the number of vehicles increases, however, it is always at least 25% also in heavy traffic conditions, and it is always significantly larger than the savings obtained by the random strategy (always at least 1/3 more, up to more than double). Regarding waiting times, as it might be expected, times increase with the number of vehicles. However, the trained bidder performs better than the random strategy, even if it does not always show a great difference. Moreover, times are always close to those of the standard strategy even if, sometimes, the latter outperforms the trained bidder, meaning that the model is able to save budget without sacrificing too much time.

Observe that experiments where the test vehicle adopts the *standard strategy* or uses the trained bidder do not present any standard deviation. This is because with no vehicle behaving randomly, the simulations are identical.

Scenario 2: percentage of random vehicles is 10%.

**Figure 2:**
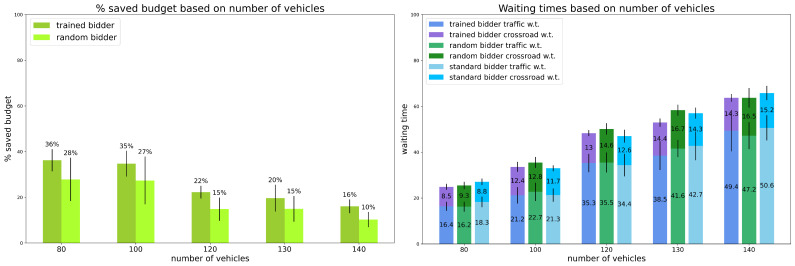
Preliminary Experiments. Results for scenario 2: percentage of random vehicles is equal to 10%. Vertical black lines represent standard deviations. Standard bidder’s savings are not reported because saved budget is always 0%.

The second model was trained using 10% of random vehicles over the total amount of vehicles. The results are reported in Figure 2.

Again, the trained bidder always saves more than the random bidder: up to 36% of its initial budget with 80 vehicles on the map, and decreasing when the number of vehicles increases (also in this case, the trained bidder is about 1/3 more efficient than the random bidder). Regarding times, the trained bidder outperforms the random one with the exception of heavy traffic conditions in which the latter is slightly faster in traffic time. However, the final trip time is comparable, as the trained bidder is faster in crossing time. The trained bidder also outperforms the standard bidder in heavy and light traffic conditions, while it is outperformed in regular traffic conditions.

Scenario 3: percentage of random vehicles is 20%.

**Figure 3:**
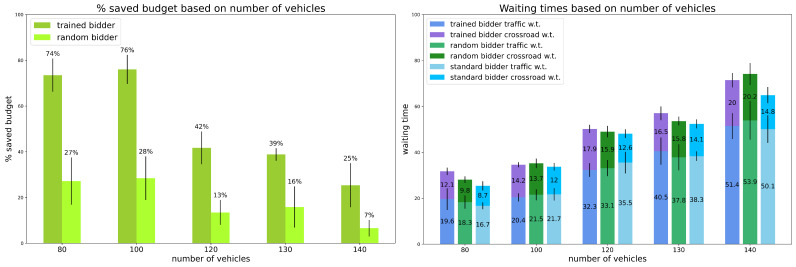
Preliminary Experiments. Results for scenario 3: percentage of random vehicles is equal to 20%. Vertical black lines represent standard deviations. Standard bidder’s savings are not reported because saved budget is always 0%.

The third model was trained using 20% of random vehicles over the total amount of vehicles. The results are reported in Figure 3.

Again, the trained bidder saves more budget than the random bidder. However, in this case, the gain is much higher than in previous configurations and huge in general: up to 76% (resp. 74%) with 100 (resp. 80) vehicles (i.e., low traffic), 42% (resp. 39%) with 120 (resp. 130) vehicles (i.e., regular traffic), and still 25% with 140 vehicles (i.e., heavy traffic). On the contrary, the random bidder slightly decreases its savings, so the trained bidder always saves almost three times what the random bidder saves.

However, when concerning waiting times, the trained bidder is outperformed by the random and the standard bidders (with the exception of the random bidder with heavy traffic), even if with small differences.

Scenario 4: percentage of random vehicles is 30%.

**Figure 4:**
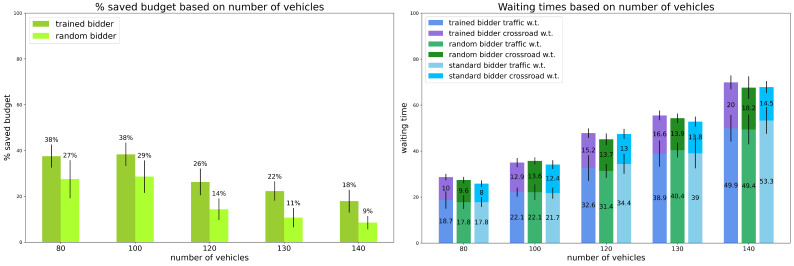
Preliminary Experiments. Results for scenario 4: percentage of random vehicles is equal to 30%. Vertical black lines represent standard deviations. Standard bidder’s savings are not reported because saved budget is always 0%.

The fourth model was trained using 30% of random vehicles over the total amount of vehicles. The results are reported in Figure 4.

Again, the trained bidder’s savings are larger than the random ones, with values that are similar to scenario 2 (also for the random bidder), in which there were 10% of random vehicles. Again, analogously to scenario 3, the trained bidder is outperformed by the random and the standard bidders for what concerns. Here the difference is slightly more evident than in the previous case, but again it is still contained.

### 3.2. Experiments

We used the model selected in preliminary experiments (i.e., trained with 20% random vehicles) to perform a set of tests to show the robustness of the proposed trained bidder under different conditions. We compared the performances of the trained bidder against the random and standard bidder as in the preliminary experiments and showed that the trained bidder is actually robust.

Experiment 1: varying the number of vehicles on the map.

**Figure 5:**
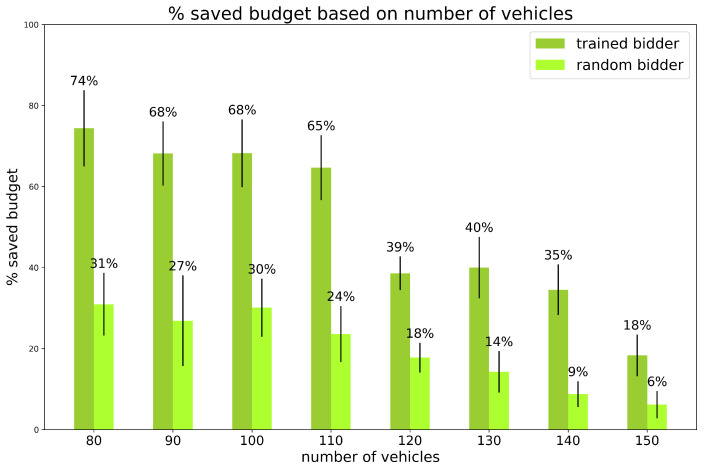
Experiments. Saved Budget for experiment 1: varying the number of vehicles, percentage of random vehicles is equal to 20%, and initial budget is fixed. Vertical black lines represent standard deviations. Standard bidder’s savings are not reported because saved budget is always 0%.

**Figure 6:**
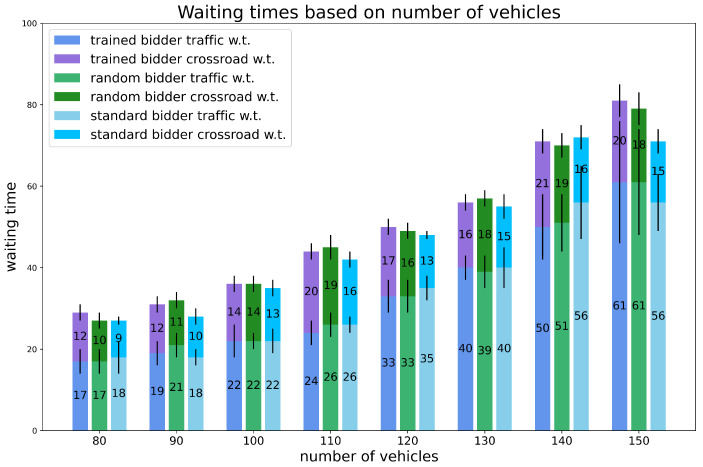
Experiments. Waiting times for experiment 1: varying the number of vehicles, percentage of random vehicles is equal to 20%, and the initial budget is fixed. Vertical black lines represent standard deviations.

We thoroughly investigated the preliminary experiment in scenario 3 by testing a finer and larger variance in number of vehicles on the map. The percentage of random vehicles is set and fixed to 20% and the initial budget is fixed to 100 for all vehicles. The results are shown in Figure 5 and Figure 6.

The results confirm that the trained bidder is capable of achieving significant savings with low traffic, but also that there is still an almost 20% savings value in very heavy traffic. In almost every case, the trained bidder’s savings are nearly 3 times those of the random bidder.

Experiment 2: varying the number of random vehicles on the map.

We thoroughly investigated the preliminary experiments by testing a different ratio of random vehicles on the map. The number of vehicles is set and fixed to 120, i.e., regular traffic conditions and the initial budget is fixed and equal for all vehicles.

**Figure 7:**
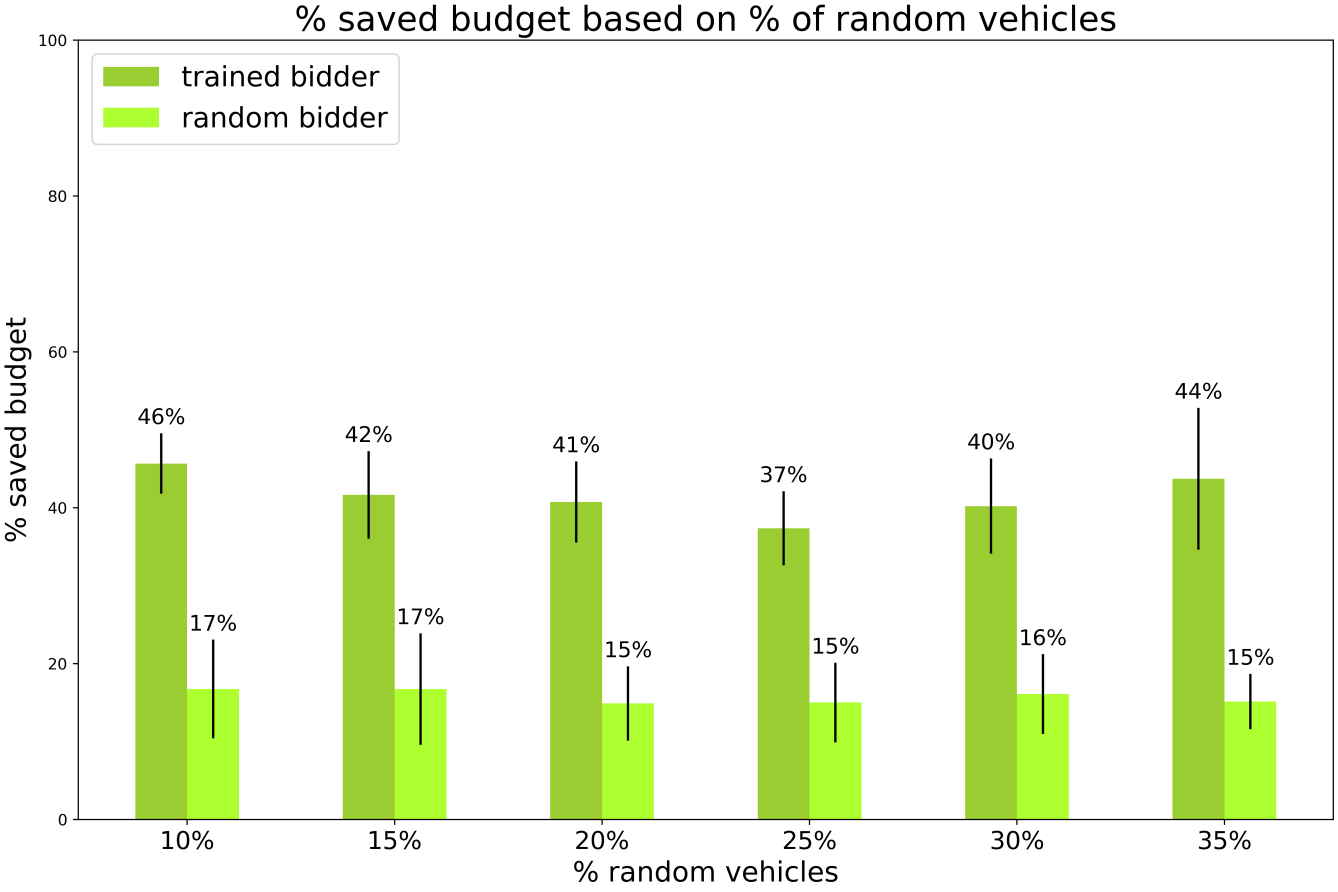
Experiments. Saved Budget for experiment 2: varying % of random vehicles, number of vehicles is set to 120 and initial budget is fixed. Vertical black lines represent standard deviations. Standard bidder’s savings are not reported because saved budget is always 0%.

**Figure 8:**
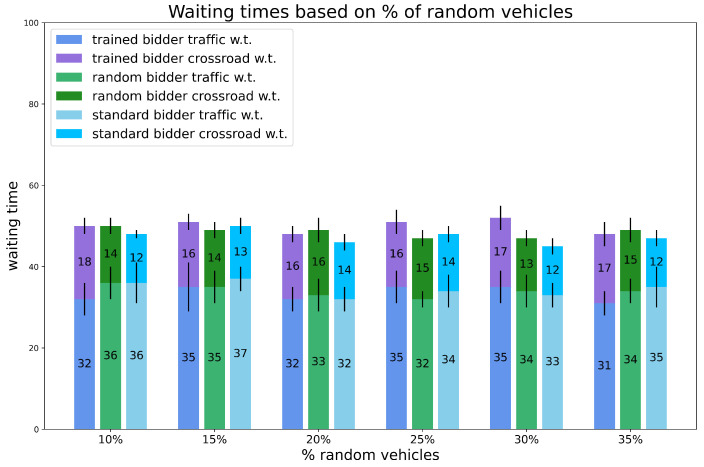
Experiments. Waiting times for experiment 2: varying % of random vehicles, number of vehicles is set to 120 and initial budget is fixed. Vertical black lines represent standard deviations.

This experiment is meant to simulate a real situation where the number of vehicles that do not perform routine paths might vary day by day in the city, so it is important that the trained bidder is robust among this type of variation. The results are shown in Figure 7 and Figure 8.

The results show that the percentage of saving does not change much when varying the number of random vehicles. The trained bidder saves at least 37% of the budget, even 46%, compared with the random bidder that saves at most 17% of the initial budget (meaning that the savings of the trained bidder are at least 2.5 times those of the random bidder). The times are comparable to competitors even if the trained bidder is often outperformed.

Experiment 3: varying the initial budget of the test vehicle.

**Figure 9:**
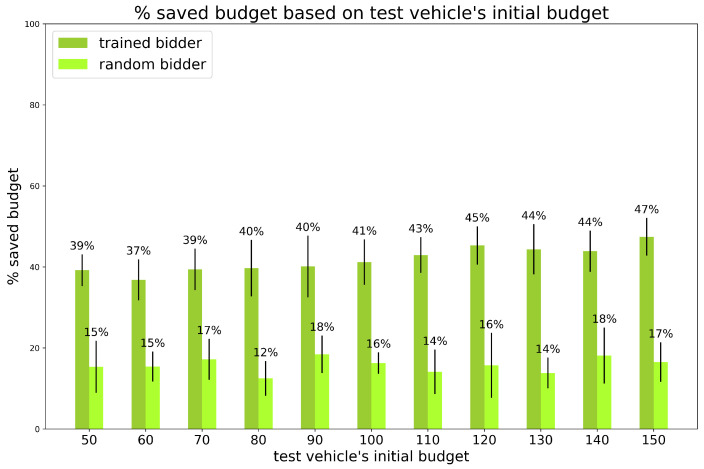
Experiments. Saved Budget for experiment 3: varying test vehicle’s initial budget, percentage of random vehicles is equal to 20% and number of vehicles is equal to 120. Vertical black lines represent standard deviations. Standard bidder savings are not reported because saved budget is always 0%.

**Figure 10:**
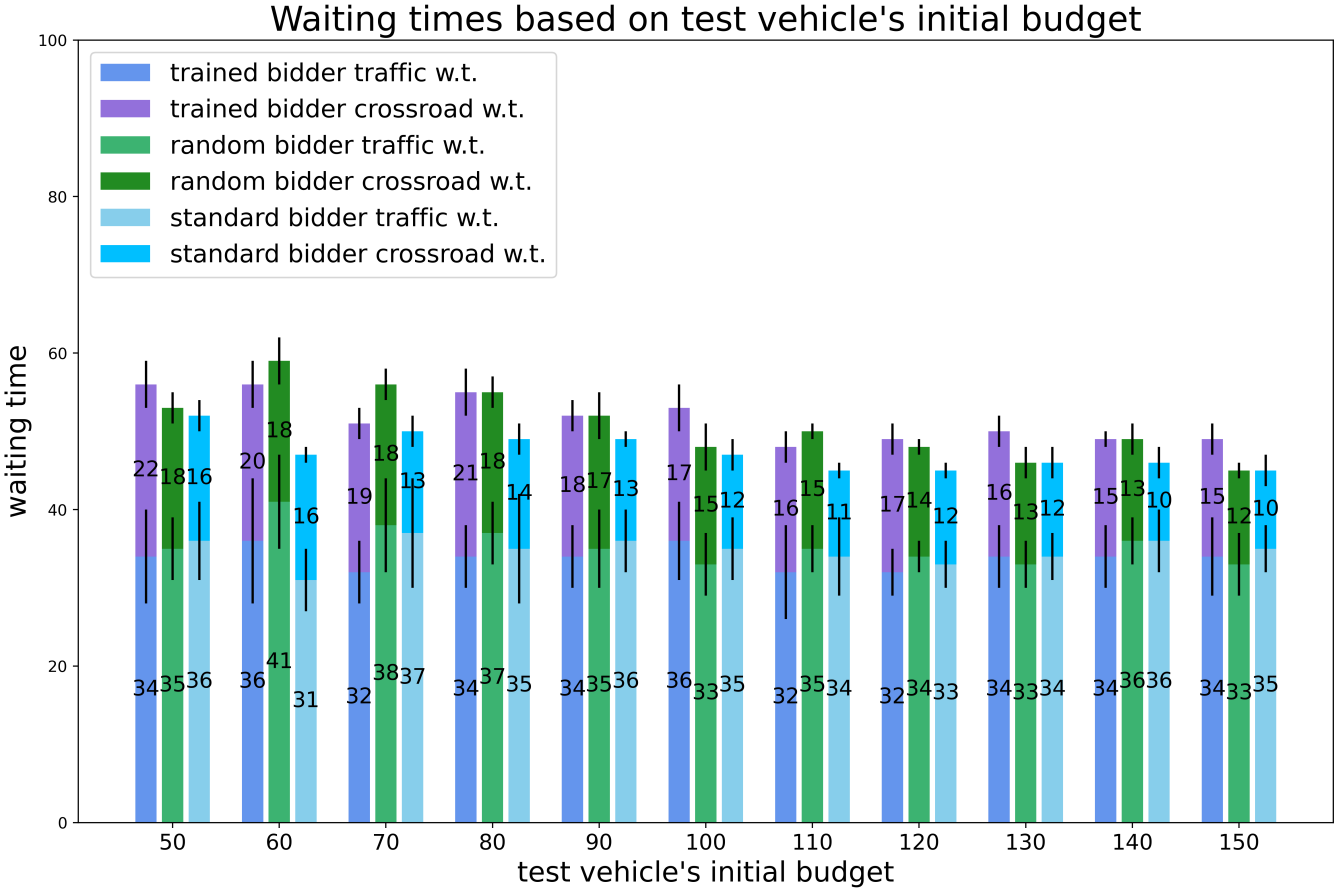
Experiments. Waiting times for experiment 3: varying test vehicle’s initial budget, percentage of random vehicles is equal to 20% and the number of vehicles is equal to 120. Vertical black lines represent standard deviations.

We varied the initial budget of the trained bidder, to simulate the situations in which the bidder has different budget values at the beginning of its trip (depending, for example, on how much of its wallet is left after several other trips). The other vehicle’s budget is fixed and equal to 100 for all. The total number of vehicles is 120 and the percentage of random vehicles is fixed to 20%. The results are shown in Figure 9 and Figure 10.

We observe that the trained bidder always saves almost 40% (in between 37% and 47%) of its initial budget (regardless of its value), outperforming the random bidder that saves no more than 18% of (nearly twice less).

Concerning times, we can see that they tend to decrease while the budget increases, as one would expect. We also see that the trained bidder is often outperformed by the others, even if the times are still comparable.

### 3.3. Discussion and Future Work

The results of the experiments show that the proposed trained bidder is able to save even huge percentages of the initial budget when vehicles travel on routine trips, at the expense of a small increase in trip times. Moreover, the model that has been trained in a given configuration is robust to variations in this configuration.

These results strongly indicate the proposal’s effectiveness, suggesting its practical adoption in the foreseen real-life scenarios, i.e., with autonomous vehicles and auction intersection management. Indeed, the trained bidder can find practical applications in everyday commuting, such as traveling between home and work/school and vice versa. This is especially beneficial for drivers who can tolerate a slight delay in reaching their destination. Note that drivers know the typical trip duration, so they can make a conscious choice. Even if traffic conditions are not exactly the same every day, there will be a sensible budget saving resulting in an overall positive trade-off between saved budget and trip time. This aspect might incentivize the adoption of the system in real-life situations, as the saved budget can be redirected for other trips, eliminating the need to earn or buy more (according to the budget distribution policy).

Furthermore, the robustness of the trained model implies that it can be trained offline through simulations, using synthetic traffic data on the driver’s city map. Subsequently, the bidder will exhibit effective performance in real-world scenarios.

This stands as a pivotal advantage for the practical implementation and adoption of the system: vehicles are not required to be equipped with powerful expensive computational devices essential for the training phase, and there is no need for continuous online training, saving valuable time. Vehicles merely require the capability to communicate with a sufficiently powerful external device. When a vehicle opts to integrate the trained bidder, it is a matter of selecting the city map and the routine trip, waiting for the simulator to complete the training (a process taking just a few minutes with the proper resources), and then putting it to use. Needless to say, the quality of the simulated data directly influences the bidder’s performance. We believe that the best method to train such models would be through running simulations on real-world maps, populated with vehicles based on traffic predictions and real-time data. The latter can be gathered by leveraging smart city infrastructures and/or vehicles themselves.

The results of our experiments, coupled with the preceding considerations, affirm that the proposed approach is worthy of further exploration. Consequently, in our future works, we intend to conduct a more extensive series of experiments to thoroughly assess the model’s capabilities and stress its limitations. Specifically, we plan to introduce increased variability, such as variations in the initial budget of the vehicles, test diverse redistribution policies of the winning bet, and incorporate real city maps into our assessments.

Moreover, our current experiments were conducted with only one test vehicle. This limitation was imposed by the considerable time and computational power required to expand our experiments to include more than one trained vehicle. As part of our future work, we aim to overcome this constraint and explore the model’s performance with multiple vehicles adopting the training bidder. Finally, it would be interesting to improve the neural network architecture, in order to forward more useful information regarding currency management to the model to be trained.

## 4. Conclusions

In this paper, we proposed a reinforcement-learning-based system that can be used by any connected vehicle to manage its budget when traveling in a smart city on a routine trip, when intersections are managed with auction-based systems. We trained various models and we have extensively tested the best-performing one in terms of balance between budget savings and waiting times. We then verified its robustness even under adverse traffic conditions.

We elaborated regarding the fact that the proposal was demonstrated to be effective and suitable for adoption in a future foreseen scenario, thus it is worth pursuing. Such a trained model might be installed on autonomous vehicles without requiring specific hardware upgrades, providing them with a smart method to manage their virtual wallet on a frequently traveled route. Future works are intended to enhance and better understand the potential of the trained bidder. In particular, these include improvements to the neural network architecture and more extensive experiments to determine the capabilities and the limitations of the system.

## Figures and Tables

**Table 1:** Summary of works for intersection management.

	Focus On	Intersection Type	Method
[5]	intersection manager	unsignalized	auctions (driver-specified budget)
[23]	intersection manager	unsignalized	auctions (two-step second-price sealed-bid)
[25]	intersection manager	unsignalized	reservation-based
[29]	intersection manager	unsignalized	reinforcement learning (to select predefined policy)
[31]	intersection manager	signalized	reinforcement learning
[33]	intersection manager	unsignalized	reinforcement learning
[34]	latencies	unsignalized	reinforcement learning
[35]	intersection manager	signalized	multi-agent reinforcement learning
[36]	vehicle manoeuvers	unsignalized	reinforcement learning
[37]	vehicle manoeuvers	unsignalized and signalized	reinforcement learning

**Table 2:** Summary of parameters related to vehicles and auctions.

Explanation	Symbol	Value
Sponsorship	β	∈[0.05,0.15]
Initial Budget	IB	∈[50…150]
Number of vehicles	VS	∈[80…150]
Maximum speed	*v*	∈[4,5,6,7]

**Table 3:** Summary of parameters, hyperparameters and thresholds.

Explanation	Symbol	Value
Reward signal	*w*	2
Penalty signal	*ℓ*	−0.3
Balancing parameter	α	0.3
Training frequency	*F*	10
Update frequency	*K*	10
Threshold	*E*	400
Batch size	*b*	32
Discount factor	γ	0.3
Learning rate	η	$10^{-4}$

## Data Availability

The data are contained within the article.
